# Supplementary material for: Community Pharmacist-Administered COVID-19 Vaccinations: A Pilot Customer Survey on Satisfaction and Motivation to Get Vaccinated
Source: Vaccines (Basel). 2021 Nov 14;9(11):1320. doi: 10.3390/vaccines9111320 (PMC8625008; doi:10.3390/vaccines9111320)
Supplement: Supplementary file 1 [file vaccines-09-01320-s001.zip › vaccines-1380182-supplementary.pdf]

vaccines

Community Pharmacist-Administered COVID-19 Vaccinations: a Pilot Customer Survey on Satisfaction and Motivation to Get Vaccinated

Supplementary Materials

Figure S1. Original survey questionnaire in German language. Translated questionnaire items (not validated): 3. I am comfortable with the COVID vaccination being done in a pharmacy. 4. I am satisfied with the injection technique at the pharmacy. 5. I am satisfied with the pre-liminary discussion of the COVID vaccination. 6. I think pharmacies are well informed about COVID vaccines. 7. I am satisfied with the premises used for COVID vaccination. 8. I would recommend COVID vaccination at the pharmacy. 9. I am satisfied with the COVID vaccine that was available. 10. I am satisfied with the way the appointment was made. 11. Is there anything you would change about the current service? 12. What do you think has already worked well?; 13. I want to reduce my own risk of contracting the coronavirus. 14. My job puts me at higher risk of contracting the coronavirus. 15. I want to protect others from being infected with the coronavirus through me. 16. I work with people who need to be protected from coronavirus infection (e.g., the elderly, the sick, nursing home residents). 17. I would like to be less restricted in everyday life. 18. I would like to be able to travel again soon. 19. I would like to follow the recommendations of the FOPH (Federal Office of Public Health). 20. I would be willing to have an annual booster vaccination (if needed). 21. I would be willing to come to the pharmacy for an annual booster vaccination (if needed). 22. I did an antibody test before deciding to vaccinate. 23. If possible, I would welcome being able to confirm the effect of the vaccination with an antibody test.; 24. Because the pharmacy was the only vaccination centre available to me. 25. Because the opening hours of the pharmacy suit me. 26. Because the pharmacy is easily accessible for me (e.g., parking, public transport connection). 27. Because I have already had good experiences with vaccinations at the pharmacy. 28. Because I don't have a general practitioner. 29. Because my physician is not available. 30. Because I trust the pharmacy. 31. Would you have had the COVID vaccination done elsewhere, e.g., by a physician, in a vaccination centre? 32. How often have you had vaccinations (including other vaccinations) at a pharmacy? 33. What is your salutation?; 34. How old are you? 35. In which zip code was the COVID vaccination carried out? 36. What is your highest acquired level of education? 37. Do you belong to a specific target risk group for COVID vaccination? 38. What vaccine did you receive today? 39. How would you describe your general vaccination coverage? 40. How often have you been vaccinated against seasonal flu (influenza) in the past few years?

Kundenzufriedenheit COVID-Impfung Apotheken Zürich

Page 1

Informationen für Teilnehmende

Vielen Dank, dass Sie sich für eine Teilnahme an unserer Studie entschieden haben. Die Auswertung Ihrer Antworten wird uns helfen, die COVID-Impfung als Dienstleistung weiter zu verbessern.

Bevor wir mit der eigentlichen Umfrage beginnen können, müssen Sie bestätigen, dass Sie die Informationen auf der Rückseite des Einladungsschreibens gelesen und verstanden haben. Dazu finden Sie unten an dieser Seite eine Bestätigungsfrage. Auch können Sie sich hier einen Nicknamen geben, den Sie uns bei einem späteren Widerruf Ihrer Einwilligung angeben können.

Bei Fragen zu dieser Studie wenden Sie sich bitte an die angegebene Kontaktperson. Die impfenden Apotheken haben aktuell keine Kapazität, Sie beim Ausfüllen dieses Fragebogens zu unterstützen.

Kontaktperson
ETH Zürich
Dr. phil. Dominik Stämpfli
eidg. dipl. pharm., FPH Klinische Pharmazie
Wissenschaftlicher Mitarbeiter Pharmakoepidemiologie
Institut für Pharmazeutische Wissenschaften
Vladimir-Prelog-Weg 4
8093 Zürich
dominik.staempfli@pharma.ethz.ch

1. Mit meinem Einverständnis zur Datenerhebung bestätige ich als teilnehmende Person:

   - Ich habe die Informationen zur Studie auf der Rückseite der Einladung gelesen und verstanden.
   - Ich erfülle die im Informationsteil genannten Bedingungen für die Teilnahme und bin mir bewusst, dass die genannten Anforderungen und Einschränkungen einzuhalten sind.
   - Ich hatte genügend Zeit, über meine Teilnahme zu entscheiden.
   - Ich nehme an der Studie freiwillig teil und bin einverstanden, dass meine Daten wie oben beschrieben verarbeitet werden.

   *

   Sind Sie mit dieser Datenerhebung einverstanden?

   ◯ Ja
   ◯ Nein

2. Sie können Ihr Einverständnis zur Bearbeitung Ihrer Daten jederzeit ohne Angabe von Gründen widerrufen. Geben Sie sich dazu bitte einen Nicknamen, den Sie uns bei Widerruf Ihres Einverständnis nennen könnten.

   Die Angabe eines Nicknamens ist freiwillig.

Kundenzufriedenheit COVID-Impfung Apotheken Zürich

Page 2

Zufriedenheit

Zu Beginn möchten wir Ihre Zufriedenheit mit der Dienstleistung erfahren. Bitte bewerten Sie, wie sehr folgende Aussagen auf Sie zutreffen. Falls Sie eine Frage nicht beantworten können - beispielsweise weil sie nicht auf Ihre konkrete Situation zutrifft - können Sie "nicht beantwortbar" auswählen.

3. Ich fühle mich wohl damit, dass die COVID-Impfung in einer Apotheke durchgeführt wird.

*

trifft eher zu

4. Ich bin zufrieden mit der Injektionstechnik in der Apotheke.

*

trifft eher zu

5. Ich bin zufrieden mit der Vorbesprechung der COVID-Impfung.

*

trifft eher zu

6. Ich denke, dass die Apotheken über die COVID-Impfstoffe gut informiert sind.

*

trifft eher zu

7. Ich bin zufrieden mit den Räumlichkeiten, welche für die COVID-Impfung verwendet werden.

*

trifft eher zu

8. Ich würde die COVID-Impfung in der Apotheke weiterempfehlen.

*

trifft eher zu

9. Ich bin zufrieden mit dem COVID-Impfstoff, der zur Verfügung stand.

*

trifft eher zu

10. Ich bin zufrieden wie die Terminvereinbarung stattgefunden hat.

*

trifft eher zu

11. Gibt es etwas, das Sie an der aktuellen Dienstleistung verändern würden?

12. Was hat Ihrer Meinung nach bereits gut funktioniert?

Kundenzufriedenheit COVID-Impfung Apotheken Zürich

Page 3

Motivation

Im Folgenden möchten wir Ihre Gründe für die COVID-Impfung erfahren. Bitte bewerten Sie, wie sehr folgende Aussagen auf Sie zutreffen. Falls Sie eine Frage nicht beantworten können - beispielsweise weil sie nicht auf Ihre konkrete Situation zutrifft - können Sie "nicht beantwortbar" auswählen.

13. Ich möchte mein eigenes Risiko reduzieren, mich mit dem Coronavirus anzustecken. *

trifft eher zu

14. Mein Beruf setzt mich einem höheren Risiko aus, mich mit dem Coronavirus anzustecken. *

trifft eher zu

15. Ich möchte andere Personen davor schützen, durch mich mit dem Coronavirus angesteckt zu werden. *

trifft eher zu

16. Ich arbeite mit Personen, welche vor einer Coronavirus-Infektion geschützt werden müssen (z. B. Betagte, Kranke, Heimbewohner). *

trifft eher zu

17. Ich möchte im Alltag weniger eingeschränkt sein. *

trifft eher zu

18. Ich möchte bald wieder verreisen können. *

trifft eher zu

19. Ich möchte den Empfehlungen des BAG (Bundesamt für Gesundheit) folgen. *

trifft eher zu

20. Ich wäre bereit, eine jährliche Auffrischimpfung (falls diese nötig sein sollte) zu machen. *

trifft eher zu

21. Ich wäre bereit, eine jährliche Auffrischimpfung (falls diese nötig sein sollte) in der Apotheke machen zu kommen. *

trifft eher zu

22. Ich habe vor der Impfentscheidung einen Antikörpertest gemacht.*

-- Please Select --

23. Falls möglich, würde ich es begrüssen, die Wirkung der Impfung mit einem Antikörpertest bestätigen zu können. *

trifft eher zu

Kundenzufriedenheit COVID-Impfung Apotheken Zürich

Page 4

Gründe für die Wahl einer Apotheke

Im Folgenden möchten wir Ihre Gründe für die Wahl einer Apotheke als Impfort erfahren. Bitte bewerten Sie, wie sehr folgende Aussagen auf Sie zutreffen. Falls Sie eine Frage nicht beantworten können - beispielsweise weil sie nicht auf Ihre konkrete Situation zutrifft - können Sie "nicht beantwortbar" auswählen.

24. Weil mir die Apotheke als einziger Impfort zur Verfügung stand.

*

trifft eher zu

25. Weil mir die Öffnungszeiten der Apotheke entsprechen.

*

trifft eher zu

26. Weil die Apotheke für mich leicht zugänglich ist (z. B. Parkplatz, Anbindung öffentlicher Verkehr).

*

trifft eher zu

27. Weil ich bezüglich Impfungen in der Apotheke bereits gute Erfahrungen gemacht habe.

*

trifft eher zu

28. Weil ich keine Hausärztin/keinen Hausarzt habe.

*

trifft eher zu

29. Weil meine Ärztin/mein Arzt nicht verfügbar ist.

*

trifft eher zu

30. Weil ich der Apotheke vertraue.

*

trifft eher zu

31. Hätten Sie die COVID-Impfung auch anderswo durchführen lassen, z.B. bei einer Ärztin / einem Arzt, in einem Impfzentrum?

*

- (•) Ja
- ( ) Nein
- ( ) Ich weiss es nicht

32. Wie oft haben Sie sich bereits in einer Apotheke impfen lassen (auch andere Impfungen)?

*

- (•) Dieses war das erste Mal
- ( ) 1-2
- ( ) 3-4
- ( ) >4

Kundenzufriedenheit COVID-Impfung Apotheken Zürich

Page 5

Allgemeine Informationen

Zum Abschluss möchten wir allgemeine Informationen erfahren. Diese helfen uns bei der Auswertung, um Zusammenhänge aufzuzeigen.

33. Wie lautet Ihre Anrede?

*

Frau

34. Wie alt sind Sie?

* The value must be between 1 and 110, inclusive.

35. In welcher Postleitzahl (PLZ) wurde die COVID-Impfung durchgeführt?

*

36. Was ist Ihr höchster erworbener Ausbildungsgrad?

Obligatorische Schule

37. Gehören Sie zu einer bestimmten Ziel-Risikogruppe für die COVID-Impfung?*

Wählen Sie bitte entsprechend aus.

-- Please Select --

38. Welchen Impstoff haben Sie heute erhalten?*

-- Please Select --

39. Wir würden Sie Ihren allgemeinen Durchimpfungsgrad bezeichnen?

*

Ich möchte die empfohlenen Basisimpfungen und ergänzenden Impfungen erhalten

40. Wie oft haben Sie sich in den vergangenen Jahren bereits gegen die saisonale Grippe (Influenza) impfen lassen?

*

- (●) 0
- ( ) 1-2
- ( ) 3-4
- ( ) >4

Table S1. Results of the Likert scale questionnaire items of individuals with complete demographic information (N = 372).

	Does not apply at all	Does not apply	Rather does not apply		Rather does apply	Does apply	Does fully apply
Choice of a pharmacy as place of vaccination							
Only place	52	131	26		76	50	30
Opening hours	22	61	35		118	75	44
Accessibility	10	24	19		79	91	146
Positive experiences	25	114	22		55	29	37
No primary care physician	76	177	21		47	32	15
Availability of physician	51	143	22		63	40	12
Trust	3	7	4		130	111	97
Satisfaction							
Preptalk	2	1	1		40	89	238
Feeling comfortable	0	0	2		19	70	281
Injection technique	1	1	1		21	46	302
Knowledge	2	0	2		29	100	234
Facility	2	1	4		53	99	213
Recommendation	2	0	3		30	70	267
Vaccine	0	3	0		54	105	196
Scheduling	5	14	15		74	70	194
Motivation							
Own risk	1	8	15		70	89	188
Job increases own risk	44	92	65		67	60	41
Others' risk	1	1	7		69	81	213
Job increases risk for others	103	125	39		60	23	18
Restrictions	0	4	6		73	88	201
Travel	5	5	15		77	85	185
Official recommendation	6	8	22		130	127	75
Antibody test post	6	36	63		123	95	43
Booster vaccination							
Yearly booster	4	11	32		106	110	107
Booster in pharmacy	0	2	13		102	67	185

Table S2. Results of the Likert scale questionnaire items in percent from individuals with complete demographic information (N = 372).

	Does not apply at all	Does not apply	Rather does not apply		Rather does apply	Does apply	Does fully apply
Choice of a pharmacy as place of vaccination							
Only place	14.25%	35.89%	7.12%		20.82%	13.70%	8.22%
Opening hours	6.20%	17.18%	9.86%		33.24%	21.13%	12.39%
Accessibility	2.71%	6.50%	5.15%		21.41%	24.66%	39.57%
Positive experiences	8.87%	40.43%	7.80%		19.50%	10.28%	13.12%
No primary care physician	20.65%	48.10%	5.71%		12.77%	8.70%	4.08%
Availability of physician	15.41%	43.20%	6.65%		19.03%	12.08%	3.63%
Trust	0.85%	1.99%	1.14%		36.93%	31.53%	27.56%
Satisfaction							
Preptalk	0.54%	0.27%	0.27%		10.78%	23.99%	64.15%
Feeling comfortable	0.00%	0.00%	0.54%		5.11%	18.82%	75.54%
Injection technique	0.27%	0.27%	0.27%		5.65%	12.37%	81.18%
Knowledge	0.54%	0.00%	0.54%		7.90%	27.25%	63.76%
Facility	0.54%	0.27%	1.08%		14.25%	26.61%	57.26%
Recommendation	0.54%	0.00%	0.81%		8.06%	18.82%	71.77%
Vaccine	0.00%	0.84%	0.00%		15.08%	29.33%	54.75%
Scheduling	1.34%	3.76%	4.03%		19.89%	18.82%	52.15%
Motivation							
Own risk	0.27%	2.16%	4.04%		18.87%	23.99%	50.67%
Job increases own risk	11.92%	24.93%	17.62%		18.16%	16.26%	11.11%
Others' risk	0.27%	0.27%	1.88%		18.55%	21.77%	57.26%
Job increases risk for others	27.99%	33.97%	10.60%		16.30%	6.25%	4.89%
Restrictions	0.00%	1.08%	1.61%		19.62%	23.66%	54.03%
Travel	1.34%	1.34%	4.03%		20.70%	22.85%	49.73%
Official recommendation	1.63%	2.17%	5.98%		35.33%	34.51%	20.38%
Antibody test post	1.64%	9.84%	17.21%		33.61%	25.96%	11.75%
Booster vaccination							
Yearly booster	1.08%	2.97%	8.65%		28.65%	29.73%	28.92%
Booster in pharmacy	0.00%	0.54%	3.52%		27.64%	18.16%	50.14%

Table S3. Results of the Likert scale questionnaire items of all filled questionnaires (N = 421), including 49 questionnaires with missing demographic information, as sensitivity analysis.

	Does not apply at all	Does not apply	Rather does not apply		Rather does apply	Does apply	Does fully apply
Choice of a pharmacy as place of vaccination							
Only place	52	133	26		80	52	30
Opening hours	22	62	36		123	76	44
Accessibility	10	25	19		84	93	146
Positive experiences	27	114	22		61	29	37
No primary care physician	78	178	22		51	32	15
Availability of physician	52	144	22		68	41	12
Trust	3	7	4		135	113	98
Satisfaction							
Preptalk	2	1	1		49	98	255
Feeling comfortable	0	0	2		25	81	299
Injection technique	1	1	1		30	52	322
Knowledge	2	0	2		38	109	251
Facility	2	1	4		60	111	229
Recommendation	2	0	3		38	78	286
Vaccine	0	3	0		67	114	209
Scheduling	5	14	18		87	76	207
Motivation							
Own risk	2	8	16		78	92	193
Job increases own risk	44	95	66		77	63	41
Others' risk	1	1	7		77	86	218
Job increases risk for others	106	125	41		72	23	19
Restrictions	0	4	6		85	90	205
Travel	5	5	15		86	88	191
Official recommendation	6	9	22		143	129	77
Antibody test post	7	36	65		134	95	46
Booster vaccination							
Yearly booster	4	11	32		116	114	110
Booster in pharmacy	0	2	13		113	69	190

Table S4. Results of the Likert scale questionnaire items of all filled questionnaires (N = 421) in percent, including 49 questionnaires with missing demographic information, as sensitivity analysis.

	Does not apply at all	Does not apply	Rather does not apply	Rather does apply	Does apply	Does fully apply
Choice of a pharmacy as place of vaccination						
Only place	13.94%	35.66%	6.97%	21.45%	13.94%	8.04%
Opening hours	6.06%	17.08%	9.92%	33.88%	20.94%	12.12%
Accessibility	2.65%	6.63%	5.04%	22.28%	24.67%	38.73%
Positive experiences	9.31%	39.31%	7.59%	21.03%	10.00%	12.76%
No primary care physician	20.74%	47.34%	5.85%	13.56%	8.51%	3.99%
Availability of physician	15.34%	42.48%	6.49%	20.06%	12.09%	3.54%
Trust	0.83%	1.94%	1.11%	37.50%	31.39%	27.22%
Satisfaction						
Preptalk	0.49%	0.25%	0.25%	12.07%	24.14%	62.81%
Feeling comfortable	0.00%	0.00%	0.49%	6.14%	19.90%	73.46%
Injection technique	0.25%	0.25%	0.25%	7.37%	12.78%	79.12%
Knowledge	0.50%	0.00%	0.50%	9.45%	27.11%	62.44%
Facility	0.49%	0.25%	0.98%	14.74%	27.27%	56.27%
Recommendation	0.49%	0.00%	0.74%	9.34%	19.16%	70.27%
Vaccine	0.00%	0.76%	0.00%	17.05%	29.01%	53.18%
Scheduling	1.23%	3.44%	4.42%	21.38%	18.67%	50.86%
Motivation						
Own risk	0.51%	2.06%	4.11%	20.05%	23.65%	49.61%
Job increases own risk	11.40%	24.61%	17.10%	19.95%	16.32%	10.62%
Others' risk	0.26%	0.26%	1.79%	19.74%	22.05%	55.90%
Job increases risk for others	27.46%	32.38%	10.62%	18.65%	5.96%	4.92%
Restrictions	0.00%	1.03%	1.54%	21.79%	23.08%	52.56%
Travel	1.28%	1.28%	3.85%	22.05%	22.56%	48.97%
Official recommendation	1.55%	2.33%	5.70%	37.05%	33.42%	19.95%
Antibody test post	1.83%	9.40%	16.97%	34.99%	24.80%	12.01%
Booster vaccination						
Yearly booster	1.03%	2.84%	8.27%	29.97%	29.46%	28.42%
Booster in pharmacy	0.00%	0.52%	3.36%	29.20%	17.83%	49.10%
